# Reappraisal of the Trophic Ecology of One of the World’s Most Threatened Spheniscids, the African Penguin

**DOI:** 10.1371/journal.pone.0159402

**Published:** 2016-07-19

**Authors:** Maëlle Connan, G. J. Greg Hofmeyr, Pierre A Pistorius

**Affiliations:** 1 DST/NRF Centre of Excellence at the Percy FitzPatrick Institute of African Ornithology, Department of Zoology, Nelson Mandela Metropolitan University, Port Elizabeth, 6031, South Africa; 2 Department of Zoology, Nelson Mandela Metropolitan University, Port Elizabeth, 6031, South Africa; 3 Port Elizabeth Museum at Bayworld, Humewood, Port Elizabeth, 6013, South Africa; Centre National de la Recherche Scientifique, Centre d'Etudes Biologiques de Chize, FRANCE

## Abstract

Many species of seabirds, including the only penguin species breeding on the African continent, are threatened with extinction. The world population of the endangered African penguin *Spheniscus demersus* has decreased from more than 1.5 million individuals in the early 1900s to c.a. 23 000 pairs in 2013. Determining the trophic interactions of species, especially those of conservation concern, is important when declining numbers are thought to be driven by food limitation. By and large, African penguin dietary studies have relied on the identification of prey remains from stomach contents. Despite all the advantages of this method, it has well known biases. We therefore assessed the African penguin’s diet, using stable isotopes, at two colonies in Algoa Bay (south-east coast of South Africa). These represent over 50% of the world population. Various samples (blood, feathers, egg membranes) were collected for carbon and nitrogen stable isotope analyses. Results indicate that the trophic ecology of African penguins is influenced by colony, season and age class, but not adult sex. Isotopic niches identified by standard Bayesian ellipse areas and convex hulls, highlighted differences among groups and variability among individual penguins. Using Bayesian mixing models it was for the first time shown that adults target chokka squid *Loligo reynaudii* for self-provisioning during particular stages of their annual cycle, while concurrently feeding their chicks primarily with small pelagic fish. This has important ramifications and means that not only pelagic fish, but also squid stocks, need to be carefully managed in order to allow population recovery of African penguin.

## Introduction

The marine environment has in recent years been profoundly altered by anthropogenic activities. Although all trophic levels have been affected, directly or indirectly, seabirds as a group have been particularly vulnerable and are among the most threatened taxonomic groups [1]. For many species, this is partially because of their obligate dependence on diminishing marine resources that are also targeted by humans (e.g. [2, 3]). Among seabirds, Spheniscidae, along with Diomedeidae, are the most threatened families [1] with more than half of the penguin species classified as vulnerable or endangered [4].

The African penguin *Spheniscus demersus* is endemic to the southern African coast and the only penguin species breeding on the African continent. Like some of its congeners in South America, the population of African penguins has declined sharply over the last century. Estimated between 1.5 and 3 million individuals in the early 1900s [5], African penguins numbered c.a. 23 000 breeding pairs in 2013 [6]. As a consequence of this rapid decline, the conservation status of African penguins was upgraded to Endangered in 2010 [7]. Reasons for the decline are multiple with historical contributors including commercial harvesting of eggs and destruction of nesting habitat. Presently, however, the most important threats are thought to be competition with industrial fisheries and climate change mediated displacement of pelagic fish prey resources [2, 5, 8].

The African penguin currently breeds at 28 localities from Hollam’s Bird Island on the Namibian coast to Bird Island in Algoa Bay on the south-east coast of South Africa [8]. Until the mid-2000s, the bulk of the African penguin population bred on the west coast of southern Africa in the Benguela current ecosystem [8]. Currently, however, more than 54% of the global population breeds in Algoa Bay within the Agulhas Bioregion, primarily on St Croix and Bird islands in Algoa Bay (7 616 and 2 843 breeding pairs in 2015, respectively; Department of Environmental Affairs, unpubl. data). A similar eastward displacement has also been observed in the sympatric Cape gannet *Morus capensis*, with the world’s largest gannetry presently found on Bird Island, Algoa Bay [6]. These trends have been linked to the eastward displacement of sardine *Sardinops sagax* and anchovy *Engraulis encrasicolus* which is thought to constitute the main prey of these two seabirds during chick rearing at various localities, including in Algoa Bay (e.g. [9–11]).

The diet of the African penguin has mainly been studied through the analysis of stomach contents obtained using the water off-loading technique (e.g. [12–14]). Stomach content analysis allows for the identification and measurements of prey remains but it can only be used at the colony during the breeding season when the birds return with full stomachs for chick provisioning. Furthermore, it is a snap-shot method as only prey ingested over the last ~12 h will be recovered [15]. To overcome these limitations in dietary studies in general, indirect methods were developed in the early 1990s. Not only do these provide information on the foraging ecology of marine top predators outside of the breeding season, but they also allow for the assessment of food assimilation in parents (rather than prey captures for provisioning purposes). The stable isotope technique is one of these [16]. Carbon and nitrogen stable isotopes are the most commonly used for the study of marine apex predators. Because of the almost conservative transfer of carbon stable isotopes from diet to consumers, carbon stable isotope values can be used to trace the carbon sources at the base of the ecosystem (e.g. nearshore vs offshore, marine vs terrestrial, benthic vs pelagic; [17, 18]). In contrast, nitrogen stable isotope values increase in a predictable manner with each trophic transfer [19, 20]. Within a given geographic area, nitrogen stable isotope values can therefore be used to determine the trophic position of predators. A further advantage of using stable isotopes is that by sampling a number of tissues with different turnover rates from the same individual, information can be collected at various time scales [21]. Whole blood is constantly renewed with a half-life estimated between 10 and 16 days depending on species (e.g. [17, 22, 23]); therefore integrating diet over the last few weeks prior to sampling. Feathers are almost pure keratin and remain isotopically inert once fully grown [17, 24]. In penguins, feather stable isotope values reflect the penguin diet of the last weeks prior to moult [25]. Another tissue that can be sampled non-destructively is the egg membranes from hatched eggs. These inform on female trophic ecology prior to or during egg formation [26, 27].

Given that the bulk of the African penguin population is now situated in Algoa Bay, and the need to understand how this species will adapt to continuing climate change and anthropogenic influences, a reassessment of their trophic ecology in the Eastern Cape is essential and timely. In this study, we used stable isotopes to investigate the trophic ecology of African penguins from St Croix and Bird islands in Algoa Bay. Due to varying energetic demands, this was done in relation to breeding stage, sex and age class. In addition to blood, feathers and egg membranes were therefore also collected to obtain information on the little known but nevertheless crucial pre-moulting and pre-laying periods.

## Materials and Methods

### Ethics statement

All fieldwork and data collection were undertaken under the research permit RES2013/18 issued by the Department of Environmental Affairs and the ethics clearance reference A13-SCI-ZOO-008 issued by the Research Ethics Committee at the Nelson Mandela Metropolitan University. Access to study sites (Bird Island and St Croix Island) and permission to conduct the research were granted by South African National Parks (SANParks), the managing authority of the islands. No penguins were injured as a result of handling and sampling for the present study. Sampling procedures were carried out briefly (<10 min of handling) by experienced researchers to minimize disturbance.

### Annual cycle of the African penguin in the Eastern Cape

Geographic variation in the timing of the annual cycle has been observed in African penguins [28]. The only detailed information for the Eastern Cape colonies [29] was derived for the period 1976 to 1982 on St Croix Island. Based on this, it appears that after a pre-moulting foraging trip that lasts an average of 35 days, birds usually start moulting in late October or in November. They complete their moult ashore over approximately 21 days. About two months following the end of the moult, the first clutches are laid. Shared incubation lasts an average of 42 days after which one or two chicks are raised over about three months by both parents. Chicks are usually classified into six categories according to plumage from P0 (newly hatched chicks) to blues (>61 days; fully grown, in full juvenile plumage without any down left) [30, 31].

### Collection of samples

**Fig 1:**
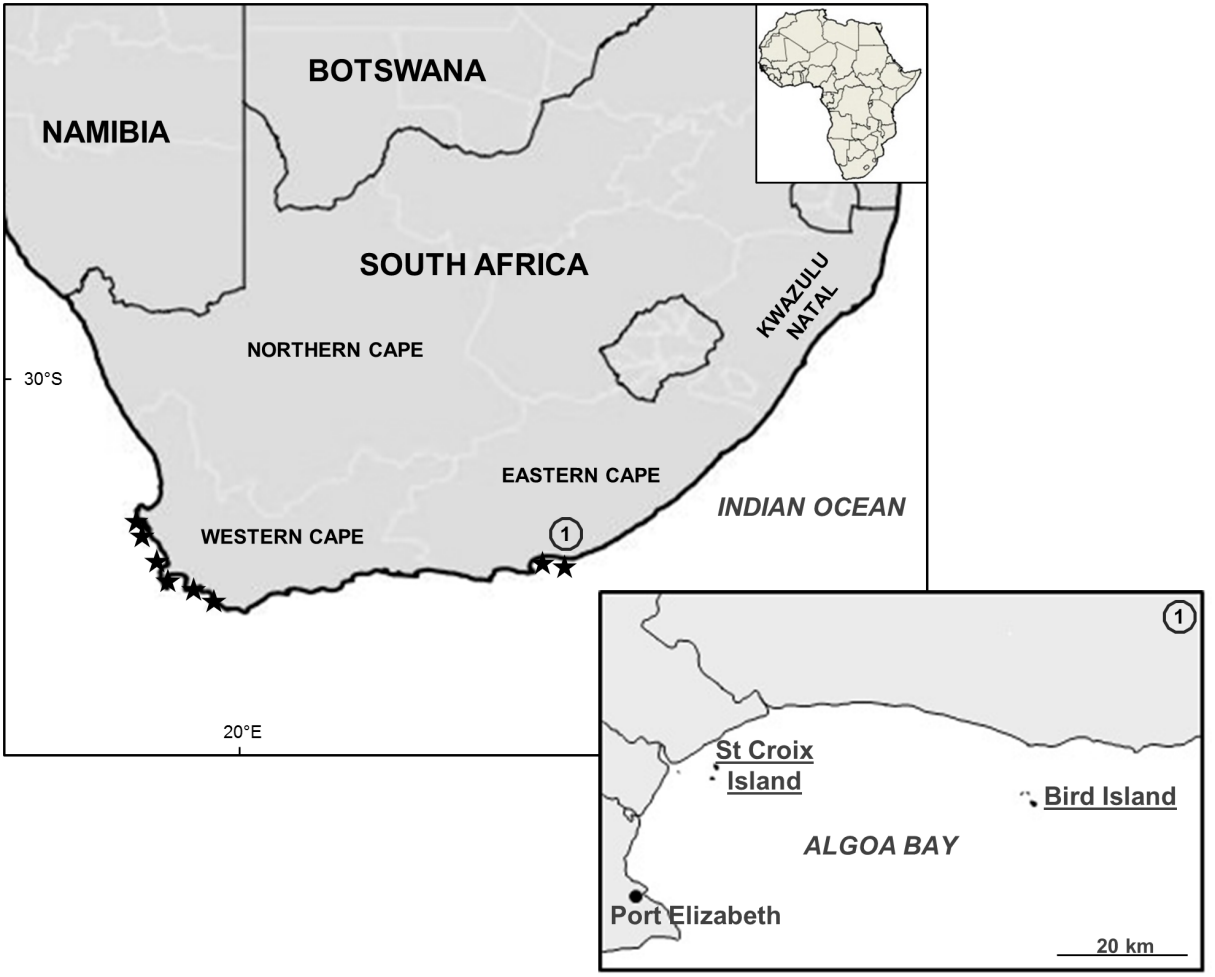
Location of the African penguin colonies along the coast of South Africa (★) and of the two colonies in the Eastern Cape where stable isotope samples were collected.

Adults and blues were sampled on St Croix and Bird islands (Fig 1) between the 23^rd^ July and 4^th^ August 2013. Adults were also caught outside their breeding season on the 19^th^ and 20^th^ January 2013 on Bird Island only, due to logistical constraints. To minimise disturbance at the nests [32], breeding adults returning from the sea close to dusk were caught on pathways leading to their nests. It was assumed that adults caught on Bird Island in January were not breeding as no eggs were found on the island over the sampling period.

For stable isotope analyses, up to 0.5 mL of blood was collected from the tarsal vein using a slightly heparinised syringe, and up to 5 white breast feathers were plucked. Egg membranes were collected from hatched eggs on both islands in April. Blood samples were kept cool before being air-dried within a few hours from collection. All samples were then stored at -20°C until further processing.

Whole blood samples were collected to inform on the diet within a month prior to sampling (i.e. mid-December/mid-January, and mid-June/July), feathers to give an indication of the diet during the pre-moulting trip (~October) and egg membrane to provide information on the pre-laying diet of females (~beginning of March) (e.g. [17, 21]).

### Structural size and body condition

Differences in stable isotope values can potentially be influenced by size and the physical condition of the African penguins. To control for this, two indices were estimated: a structural size index and a body condition index.

A number of measurements were taken from each adult and blue including bill length and depth for sexing in the field [33], flipper length and body mass. As bill length, bill depth and flipper length were correlated in adults (Pearson’s r, all P < 0.001), a principal component analysis was used to establish an index of structural size [34]: Principal Component 1 = 0.59 * bill length + 0.61 * bill depth + 0.53 * flipper length. The first principal component analysis explained 69% of the variability in the data. Body condition of adults was then defined as the residuals of a regression of body mass on the index of structural size ([34, 35]; R^2^ = 0.26, P < 0.001). Similarly, an index of structural size was defined for blues: Principal Component 1 = 0.60 * bill length + 0.54 * bill depth + 0.59 * flipper length. Their body condition was defined as the residuals of a regression of body mass on the index of structural size (R^2^ = 0.55, P < 0.001).

### Genetic sexing of African penguins

As one of the aims of the study was to examine whether differences in diet existed between sexes, assigning sex was crucial. Using length and depth measurements of the bill to build a discriminant function, >90% of the adults can be sexed accurately [33]. This leaves ~10% of penguins with borderline measurements with uncertain identification of sex. Therefore, all adults were sexed genetically by adapting a protocol developed for Cape gannets [36, 37].

Whole genomic DNA was extracted from the feather roots using a Chelex^®^ extraction method [37]. The DNA yield was then measured using a NanoDrop^®^ Spectrophotometer (Thermo Scientific), and the supernatant stored at -20°C. DNA fragments of the sex-linked CHD-1 gene (ZZ for males, ZW for females) were then amplified as in [37]. The only difference with the published protocol was the electrophoresis conditions for the separation of the PCR products which were 100 V for 1 h. Gel was then stained with GelRed^™^ Nucleic Acid Gel stain and bands were visualized under ultraviolet light.

### Stable isotope analyses

Prior to analysis, egg membranes were carefully brushed in distilled water before being dried at 50°C for 24 hours. Feathers were initially washed in chloroform-methanol (2 parts to 1) in an ultrasonic bath for 2 minutes, rinsed in successive baths of methanol and distilled water, and then dried at 50°C for 24 hours. Each feather and piece of membrane were then cut into small pieces and homogenized. For whole blood sampled from birds, it is usually assumed that no pre-treatment is necessary [38]. Homogenized feather, egg membrane and dried blood were then weighed out 0.4 to 0.5 mg into a tin capsule. Relative isotope abundances of carbon and nitrogen were determined with a Thermo Finnigan Delta XP Plus mass spectrometer interfaced with a Conflo III device to a Thermo Flash EA 1112 elemental analyzer (Stable Light Isotope Unit, University of Cape Town, South Africa). Carbon and nitrogen results are presented in the usual δ notation relative to Vienna Pee Dee Belemnite, and atmospheric N_2_, respectively. Internal laboratory standards were calibrated against reference materials from the International Atomic Energy Agency (IAEA, Vienna, Austria) and run throughout all runs, typically 2 standards for every 10–12 samples. Within and among run measurement errors are detailed in S1 Table.

Within an organism, lipids are typically depleted in ^13^C and exhibit more negative δ^13^C values than proteins and carbohydrates [39, 40]. A strong relationship between lipid contents and C:N ratios has been highlighted in animal tissues and it is advisable to account for lipids when C:N ratios are above 3.5 [41]. Accordingly, when C:N ratios exceeded 3.5 in blood samples, lipid-associated biases of δ^13^C were therefore reduced by mathematically normalizing the carbon ratios using an equation developed for aquatic animals δ^13^C = δ^13^C − 3.32 + (0.99 x C:N) [41].

### Stable isotope Bayesian ellipses

After verification of the multivariate normality assumptions (Mardia tests; all P > 0.05), stable isotope Bayesian ellipses were generated in R ([42]; package SIBER in R v3.2.5; R Foundation for Statistical Computing, Vienna, Austria) to evaluate the variability among individuals and plot the isotopic niches of the various sample groups. The following metrics were estimated: convex hull [43], standard ellipse area corrected (SEAc) for low sample size (SEAc = SEA(n-1)(n-2)^-1^), and the Bayesian estimate of the standard ellipse area (SEA_B_) [42].

### Diet reconstruction using Bayesian mixing models

In all mixing models, determining stable isotope values of potential prey species is necessary. Fifteen specimens of the five potential prey species (4 fish and 1 squid) previously identified for African penguins in Algoa Bay [14], were collected from local fisheries operating in Eastern Cape waters in 2013: anchovy, sardine, red-eye round herring *Etrumeus whiteheadi*, chub mackerel *Scomber japonicus* and squid *Loligo reynaudii*. A muscle section was sampled from each individual, dried at 50°C for 24 h, ground to powder and delipidated using cyclohexane (protocol detailed in [44]). Carbon and nitrogen stable isotope values of the prey species were correlated (Spearman’s rank coefficient P < 0.002) and failed the hypothesis of multivariate normality (Mardia’s skewness test P < 0.001). The comparison among the stable isotope values of prey species was performed using a one-way permutational analysis of variance (PERMANOVA).

Accurate diet-tissue discrimination factors are also essential in mixing models [45]. A feeding captivity study conducted with African penguins found a discrimination factor for nitrogen for whole blood only (+2.5 ± 0.2 ‰; [46]). To date, no other discrimination factors exist for African penguins. Additional average discrimination factors for carbon and nitrogen were consequently calculated from all studies conducted on other species of penguins in captivity (S2 Table). The following discrimination factors were used for all the sources: whole blood (carbon: -0.4 ± 0.6 ‰, nitrogen: +2.4 ± 0.3 ‰), feathers (carbon: +0.5 ± 0.7 ‰, nitrogen: +4.1 ± 0.7 ‰), and egg membranes (carbon: +2.8 ± 0.1 ‰, nitrogen: +4.4 ± 0.1 ‰).

The adequacy of the food sources and discrimination factors was checked using simulated mixing polygons [47] before running the mixing models. Probability distributions for the proportional contribution of the five potential prey species to the diet of African penguins was then estimated using the Bayesian stable isotope mixing model MixSIAR GUI v3.0 [48, 49]. This Bayesian approach accounted for the uncertainty in sources [50, 51], and the inclusion of categorical covariates (sexes and age classes) into the models where appropriate [48, 52]. Concentration dependence can also be included in this model [49]; but because all five potential prey species exhibited similar carbon and nitrogen concentrations (carbon: ~43%; nitrogen: ~13%) this factor did not need to be accounted for [45, 53]. Markov Chain Monte Carlo parameters for blood and feathers were set as follows: chain length = 100 000, burn in = 50 000, thin = 50, number of chains = 3. For egg membranes, parameters were chain length = 300 000, burn in = 200 000, thin = 100, number of chains = 3. Models were assessed for convergence using Gelman-Rubin and Geweke metrics [49].

### Statistical analyses

All statistical analyses were conducted using R v3.2.3 [54] and Past 3.06 [55]. Significance level was set at 0.05 and a Bonferroni correction was applied after multiple comparisons. After checks for normality and homoscedasticity, morphometric data were analysed using parametric analyses. Growth and nutritional stress may affect stable isotope values [25, 56–58]. The influence of body condition on δ^13^C and δ^15^N values were therefore checked within each group (considering locations, age classes and seasons) by comparing values for individuals exhibiting a lower (negative) and higher (positive) body condition index than average. The comparison of stable isotope values between sexes, age classes and islands required the use of various parametric and non-parametric analyses depending on whether normality and homoscedasticity hypotheses were verified.

## Results

Overall, males exhibited statistically larger features and were heavier than females (all P < 0.001; Table 1, S3 Table). A comparison of birds from the two islands found no statistical differences in morphometric data, body weight or body condition index (Table 1, S3 Table). Body condition did not influence δ^13^C in adults or in blues (all P > 0.341). Similarly, no statistical differences were found in the δ^15^N values between birds with different body conditions (all P > 0.204) with the exception of adults sampled outside the breeding season on Bird Island. In the latter case, birds with a lower body condition than average exhibited statistically higher δ^15^N values (t-test t = 2.42, P = 0.027).

**Table 1:** Biological characteristics of the African penguins sampled on Bird and St Croix islands in 2013.

Island	Period of sampling	Age class	Sex	n	Bill depth (mm)	Bill length (mm)	Flipper length (mm)	Body weight (kg)	Body condition index
**Bird**	**Non-breeding**	**Adults**	**Males**	10	25.1 ± 1.2	60.5 ± 3.0	191 ± 8	4160 ± 348	232 ± 324
					(23.6–27.3)	(57.1–66.8)	(180–206)	(3625–4625)	(-319–663)
			**Females**	12	23.2 ± 1.0	56.4 ± 2.1	181 ± 6	3735 ± 477	107 ± 498
					(21.3–24.3)	(53.4–59.3)	(170–191)	(2875–4375)	(-802–727)
	**Breeding**	**Adults**	**Males**	13	24.7 ± 0.8	60.2 ± 1.8	190 ± 7	3810 ± 309	-86 ± 340
					(23.5–26.8)	(57.4–63.2)	(176–199)	(3250–4250)	(-781–322)
			**Females**	9	22.3 ± 1.0	56.1 ± 2.0	177 ± 8	3314 ± 408	-216 ± 394
					(20.3–23.9)	(53.0–59.0)	(163–189)	(2775–4100)	(-702–398)
		**Blues**		12	17.8 ± 1.0	48.1 ± 3.9	183 ± 11	3100 ± 354	-20 ± 207
					(15.6–19.4)	(41.7–53.5)	(167–204)	(2650–3675)	(-391–241)
**St Croix**	**Breeding**	**Adults**	**Males**	13	25.2 ± 1.3	59.5 ± 2.1	185 ± 11	3873 ± 217	79 ± 255
					(23.0–27.8)	(55.4–62.3)	(167–204)	(3425–4200)	(-268–429)
			**Females**	7	22.1 ± 0.5	55.7 ± 2.8	176 ± 3	3293 ± 314	-194 ± 310
					(21.4–22.6)	(50.3–59.1)	(172–180)	(2875–3725)	(-692–152)
		**Blues**		11	17.1 ± 0.9	48.5 ± 2.2	177 ± 8	3002 ± 352	22 ± 257
					(15.2–17.9)	(45.4–52.4)	(160–185)	(2550–3750)	(-415–579)

n: number of individuals sampled. Values are means ± SD.

### Seasonal variation in carbon and nitrogen stable isotope values of blood and feathers

Carbon stable isotope values of blood collected during the non-breeding period were statistically lower in females than in males. These values for females were also lower than those for both sexes sampled during the breeding period at the same island (Kruskal-Wallis H = 22.38, P < 0.001; Mann-Whitney pairwise comparisons all P < 0.020 when considering blood from females compared to other groups). Importantly, these carbon values were correlated with high C:N ratios (up to 4.3; Pearson’s r = -0.90, P < 0.001), while C:N values were all lower than 3.5 for all the other samples. Consequently, non-breeding female blood δ^13^C were normalized and only δ^13^C_normalized_ for the females was used for subsequent analyses.

**Fig 2:**
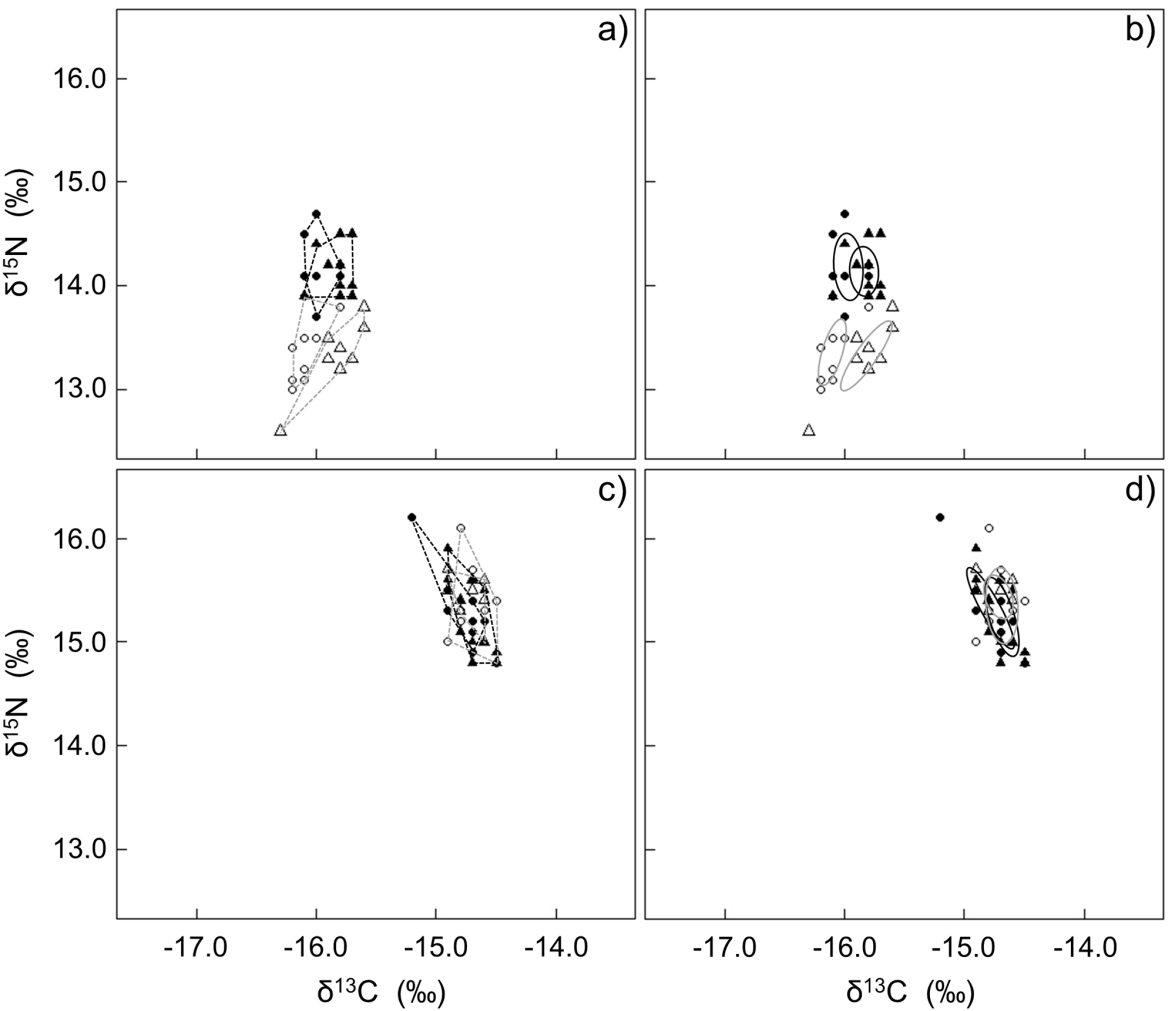
Seasonal variation in the isotopic space depicting niche areas for African penguin blood (a, b) and feathers (c, d) using convex hull areas (a, c; [31]) and standard ellipse areas corrected for small sample size (b, d; [30]). Females (circles) and males (triangles) were sampled in both non-breeding and breeding periods on Bird Island and are represented in black and grey, respectively.

Significant seasonal variations were evident based on blood samples collected during breeding and non-breeding (MANOVA Wilks’ lambda F_6,70_ = 17.73, P < 0.001, pairwise comparison all P < 0.004 with the exception of breeding season samples where no difference existed between male and female samples P = 0.391; Table 2). Samples collected outside the breeding season were segregated from breeding season samples by their δ^15^N values (t-test t = -9.26, P < 0.001), and during non-breeding males were separated from females based on their δ^13^C values (t-test t = -2.57, P < 0.020). The overall isotopic spaces of all four groups were similar (Table 3) and no overlaps were found between SEAc of non-breeding males, non-breeding females and both sexes during the breeding season (Fig 2a and 2b).

**Table 2:** Carbon (a) and nitrogen (b) stable isotope (‰), and C:N ratio (c) values of blood, feathers and egg membranes of African penguins breeding in Algoa Bay.

	Bird Island					St Croix Island		
	Non-breeding		Breeding			Breeding		
	Adults		Adults		Blues	Adults		Blues
	Males	Females	Males	Females		Males	Females	
**a) δ**^**13**^**C (‰)**								
Blood	-15.8 ± 0.2	-16.1 ± 0.1*	-15.8 ± 0.1	-16.0 ± 0.1	-15.9 ± 0.1	-15.8 ± 0.1	-15.8 ± 0.1	-15.8 ± 0.2
Feathers	-14.7 ± 0.1	-14.7 ± 0.1	-14.7 ± 0.1	-14.8 ± 0.2	-14.6 ± 0.1	-14.8 ± 0.1	-14.8 ± 0.1	-14.6 ± 0.1
Egg membrane	-	-	-	-14.6 ± 0.1	-	-	-14.6 ± 0.1	-
**b) δ**^**15**^**N (‰)**								
Blood	13.3 ± 0.3	13.4 ± 0.3	14.1 ± 0.2	14.2 ± 0.3	13.4 ± 0.2	14.1 ± 0.2	14.1 ± 0.1	13.8 ± 0.2
Feathers	15.4 ± 0.2	15.4 ± 0.4	15.3 ± 0.4	15.3 ± 0.4	14.3 ± 0.2	15.3 ± 0.2	15.4 ± 0.3	14.5 ± 0.2
Egg membrane	-	-	-	14.7 ± 0.3	-	-	14.9 ± 0.4	-
**c) C:N**								
Blood	3.3 ± 0.0	3.8 ± 0.3	3.4 ± 0.1	3.4 ± 0.1	3.4 ± 0.1	3.3 ± 0.1	3.3 ± 0.1	3.4 ± 0.1
Feathers	3.1 ± 0.0	3.1 ± 0.0	3.1 ± 0.0	3.1 ± 0.0	3.1 ± 0.0	3.1 ± 0.0	3.1 ± 0.0	3.1 ± 0.0
Egg membrane	-	-	-	3.2 ± 0.1	-	-	3.2 ± 0.1	-

*: corrected values due to high C:N values (see text). Values are means ± SD.

**Table 3:** Average standard ellipse areas (‰²) with 95% confidence intervals estimated from δ^13^C and δ^15^N values using a Bayesian Inference with 10,000 replications.

Island	Season	Age class	Sex	Blood		Feather		Egg membrane	
				SEA_B_		SEA_B_		SEA_B_	
**Bird**	**Non-breeding**	**Adults**	**Males**	0.1	(0.1–0.2)	0.1	(<0.1–0.1)	-	-
			**Females**	0.1	(<0.1–0.2)	0.1	(0.1–0.3)	-	-
	**Breeding**	**Adults**	**Males**	0.1	(<0.1–0.2)	0.1	(0.1–0.2)	-	-
			**Females**	0.1	(<0.1–0.2)	0.1	(0.1–0.3)	0.1	(0.1–0.3)
		**Blues**		0.1	(<0.1–0.2)	0.1	(<0.1–0.1)	-	-
**St Croix**	**Breeding**	**Adults**	**Males**	0.1	(<0.1–0.1)	0.1	(<0.1–0.1)	-	-
			**Females**	< 0.1	(<0.1–0.1)	0.1	(<0.1–0.2)	0.2	(0.1–0.3)
		**Blues**		0.1	(0.1–0.2)	0.1	(<0.1–0.1)	-	-

No seasonal or sex related variation in δ^13^C and δ^15^N were highlighted in the feather samples (PERMANOVA F_season_ = 1.04, P = 0.296; F_sex_ = 0.06, P = 0.894; Tables 2 and 3) with all the SEAc and TA overlapping (Fig 2c and 2d). Similarly, their interaction was not significant either (F_season*sex_ = -2.96, P = 0.583).

### Spatial variation in carbon and nitrogen stable isotope values of the three tissues

**Fig 3:**
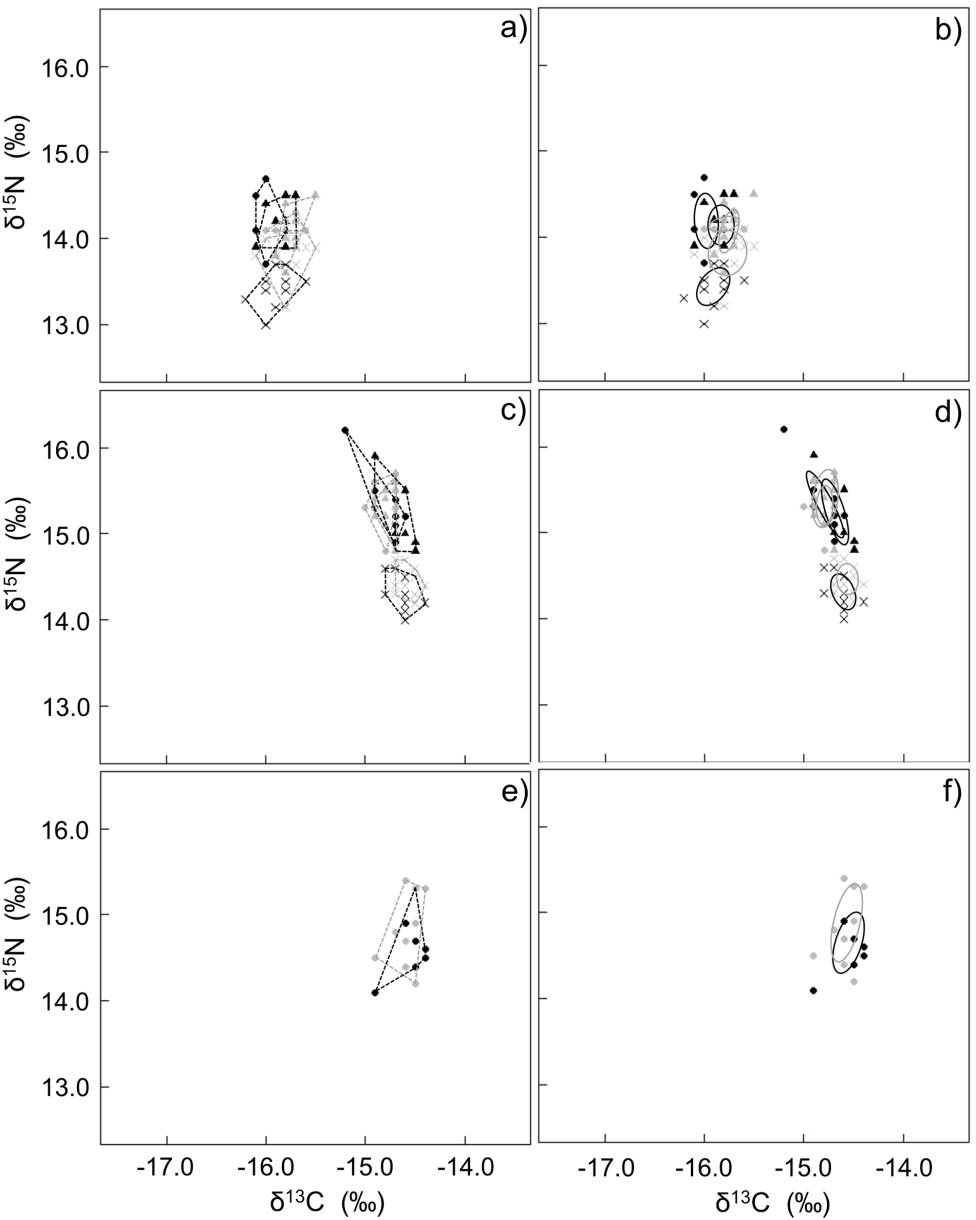
Spatial variation in the isotopic space depicting niche areas for African penguins blood (a, b), feathers (c, d) and egg membrane (e, f) using convex hull areas (a, c, e; [31]) and standard ellipse areas corrected for small sample size (b, d, f; [30]). Females (circles), males (triangles) and blues (crosses) sampled on Bird and St Croix islands are represented in black and grey, respectively.

When using a multivariate analysis, neither sex, island nor their interaction had a significant effect on adult blood stable isotope values (PERMANOVA, F_island_ = 2.63, P = 0.077; F_sex_ = 2.02, P = 0.116; F_island*sex_ = -2.71, P = 0.736). Univariate analysis however showed that carbon stable isotope values of adult blood samples were significantly different between the two islands with samples from St Croix being depleted in ^13^C (ANOVA F_island_ = 11.68, P < 0.002; Table 2). Sex was also a significant factor with females exhibiting a lower δ^13^C than males (ANOVA F_sex_ = 7.76, P < 0.009; Table 2). The interaction between those two factors was not significant (ANOVA F_island*sex_ = 1.23, P = 0.274). None of these factors were significant when considering δ^15^N values (ANOVA F_island_ = 0.56, P = 0.458; F_sex_ = 0.74, P = 0.394; F_island*sex_ = 0.05, P = 0.817). When adding the samples for blues, the island still had a significant effect on δ^13^C values (ANOVA F_island_ = 13.57, P < 0.001; Table 2) but age class did not (ANOVA F_age class_ = 0.07, P = 0.791), and neither did the interaction of these two factors (ANOVA F_island*age class_ = 0.06, P = 0.814; Fig 3a and 3b). Conversely, δ^15^N values from blues were significantly lower than the values from adults (ANOVA F_age class_ = 71.89, P < 0.001), and the interaction island/age class had a significant impact on δ^15^N values (ANOVA F_island*age class_ = 13.75, P < 0.001; Fig 3a and 3b).

Neither sex, island nor their interaction had a significant influence on the δ^13^C and δ^15^N adult feather values (PERMANOVA F_island_ = 0.50, P = 0.504; F_sex_ = 0.77, P = 0.383; F_island*sex_ = -1.95, P = 0.661). When considering samples from adults and blues, no statistical difference in the stable isotope values was highlighted between the two islands, but age class was a significant factor (PERMANOVA F_island_ = 1.23, P = 0.240; F_age class_ = 117.09, P < 0.001) with δ^13^C and δ^15^N values being significantly lower in blues than in adults (δ^13^C: Mann-Whitney U = 152, P < 0.001; δ^15^N: Mann-Whitney U = 0, P < 0.001; Table 2; Fig 3c and 3d).

No statistical differences were apparent in egg membranes from the two islands neither for δ^13^C (Mann-Whitney U = 45, P = 0.723) nor for δ^15^N (t test t = -1.33, P = 0.199; Table 2). Finally, the isotopic space from both islands was similar (Table 3; Fig 3e and 3f).

### Diet reconstruction

δ^13^C and δ^15^N values of the five marine species, sardine, anchovy, red-eye round herring, chub mackerel and squid, were significantly different from each other (PERMANOVA F_4,70_ = 243.2, P < 0.001, pairwise comparisons all P < 0.02; S4 Table) and were therefore integrated into the mixing model without grouping.

**Fig 4:**
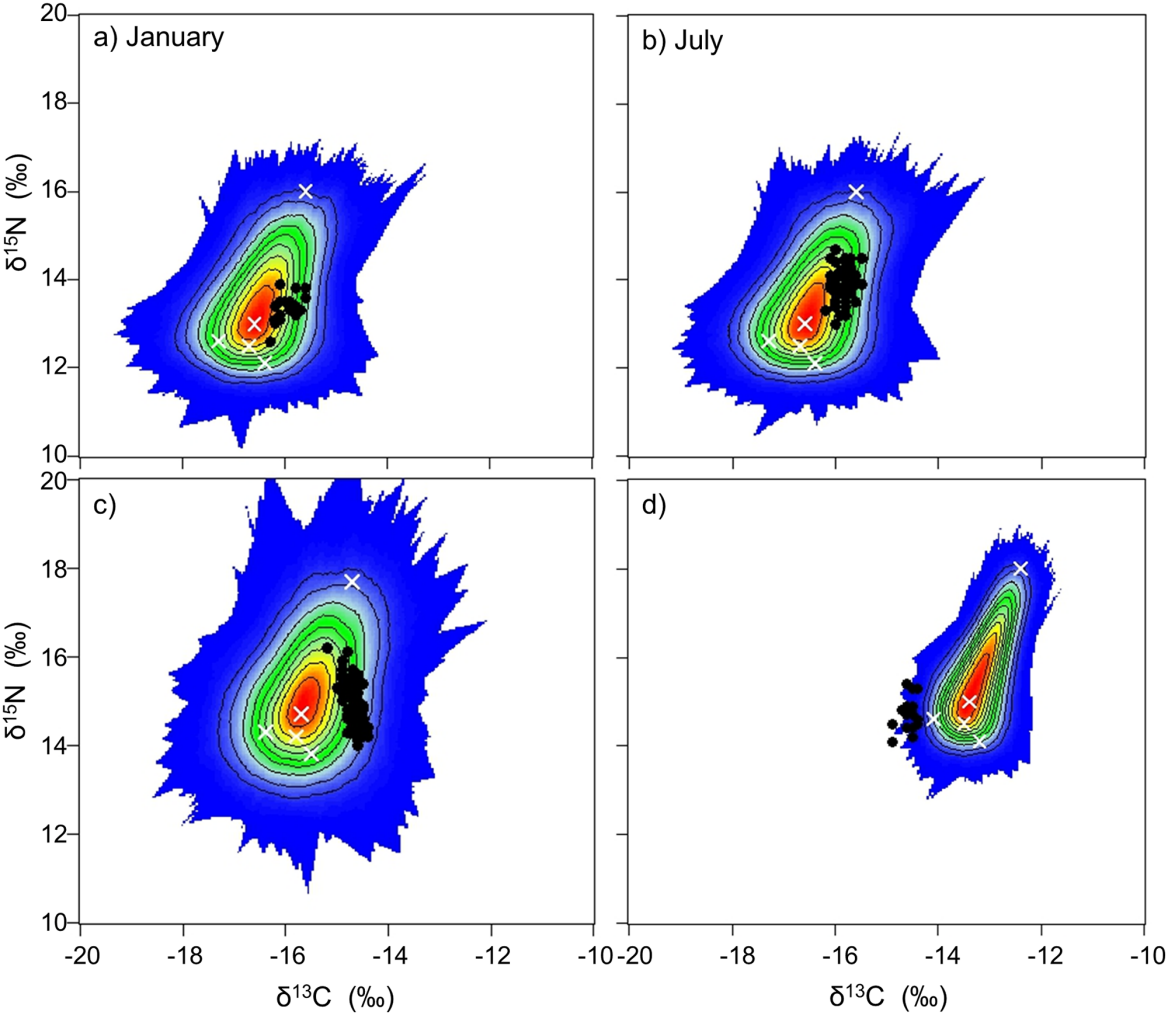
Simulated mixing region calculated with the five potential prey species (white crosses). Dark symbols represent the African penguin blood (a and b), feather (c) and egg membrane (d) data corrected with the tissue-specific trophic enrichment factors (see S2 Table).

When corrected with tissue-specific trophic enrichment factors, African penguin δ^13^C and δ^15^N values fell within the simulated mixing polygons calculated with the five potential prey species (Fig 4) allowing further diet determination using Bayesian mixing models. Egg membrane δ^13^C and δ^15^N values were, however, borderline to the simulated mixing polygons.

**Fig 5:**
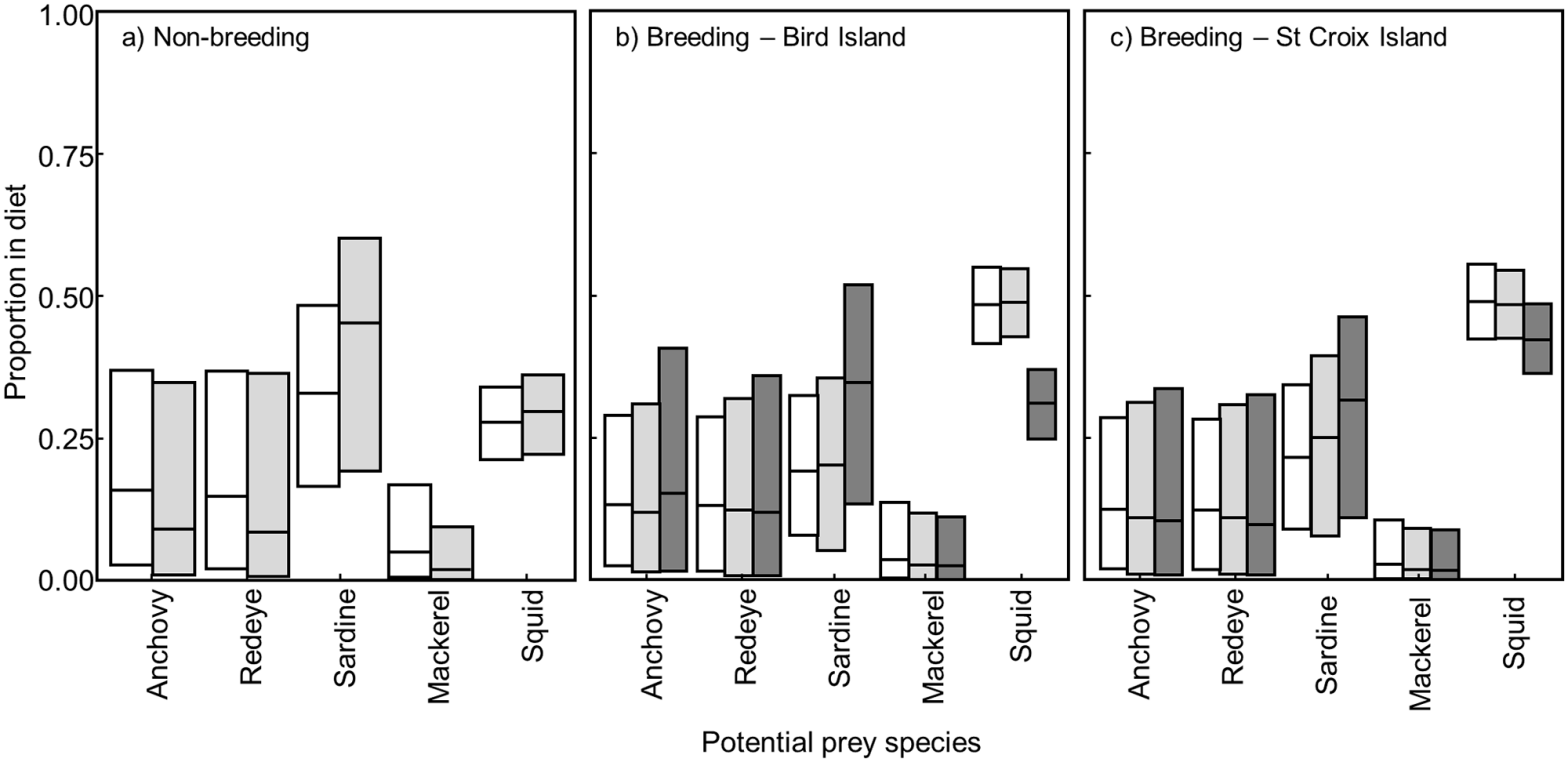
Stable isotope mixing model (MixSIAR) results with predicted diet proportions (median values and 5^th^ to 95^th^ percentiles) of each five potential prey species compared to δ^13^C and δ^15^N values of African penguin blood. White: females, light grey: males, dark grey: blues.

**Fig 6:**
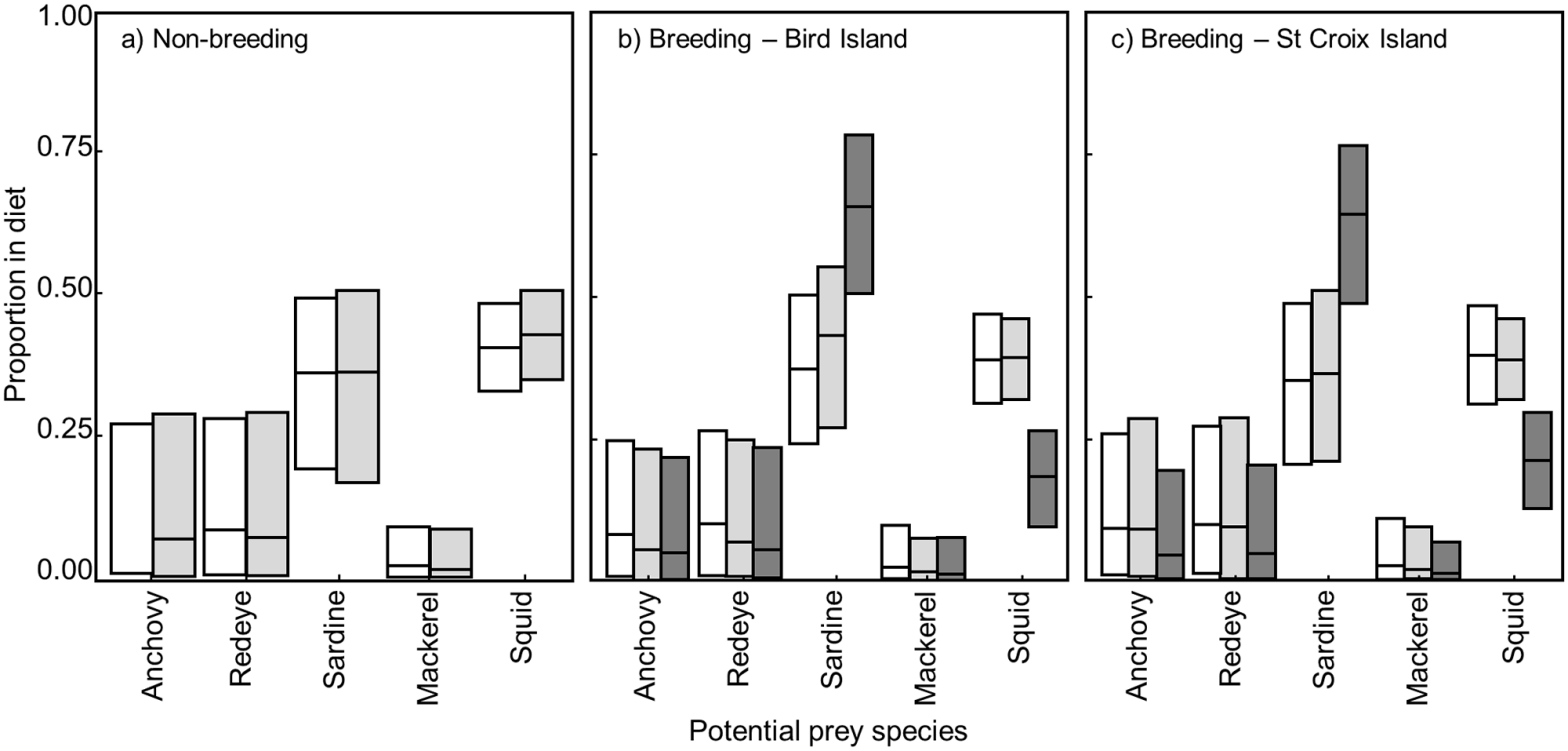
Stable isotope mixing model (MixSIAR) results with predicted diet proportions (median values and 5^th^ to 95^th^ percentiles) of each five potential prey species compared to δ^13^C and δ^15^N values of African penguin feathers. White: females, light grey: males, dark grey: blues.

Unlike the data for blood and feathers, MixSIAR models did not converge for the egg membrane data, which was therefore not used for diet reconstruction. Overall, models predicted that sardine and squid were the main species eaten by the various groups of birds (Figs 5 and 6; S5 Table). No sex-specific differences in the diet of penguins were apparent. Blood data suggested that adults from Bird Island mainly preyed upon sardine, and to a less extent squid, before the breeding season with squid consumption becoming dominant during the breeding season. Similarly, squid were the main prey species for breeding birds from St Croix. Prior to moulting, adults from both islands favoured a mix of sardine and squid.

Blood samples collected from both adults and blues on Bird Island indicated that while mainly utilising squid themselves, breeding birds raised their chicks primarily with sardine followed by squid. Feather samples from blues confirmed this result. On St Croix, blues were again mainly raised on sardines but squid quantities were slightly higher than for blues on Bird Island.

Importantly, due to a correlation between sardine and anchovy stable isotope values, in many cases the model could not fully discriminate between those two species. Therefore, a grouping of ‘small pelagic fish’ may be a more realistic diet component rather than strictly sardine *sensus stricto*.

## Discussion

The African penguin population has decreased dramatically due to a number of factors. Currently, this includes shortages in prey [8]. To fully understand and assess how present and potential future changes in the marine environment will affect this species, it is crucial to accurately determine its trophic interactions and whether these vary according to intrinsic (age classes, sexes) but also extrinsic (seasons, colonies) factors. This study addresses some of these concerns and for the first time shows that season, colony as well as age class affect stable isotope data in African penguins through their diet. It furthermore demonstrates that the contribution of squid in the African penguin diet has largely been overlooked in previous studies that only relied on stomach content analyses. Our study also stresses the importance of indirect methods in adequately determining the diet of marine top predators at a range of spatial scales and life history stages. This is particularly important where appropriate management of prey resources throughout the annual cycle could be critical to the future well-being of the species.

### Potential effect of the annual cycle of the African penguin on carbon and nitrogen stable isotope values

The annual cycle of penguins includes two important events, moulting and breeding, each being energetically costly in its own way [59]. Prior to the moulting fast ashore, penguins maximize energy intake to increase fat reserves. During breeding, breeders face the costs of incubation and raising chicks, in addition to self-maintenance [60]. Body weight and condition indices suggested that African penguin males are truly income breeders, i.e. they acquire the necessary energy to raise their offspring from prey caught while rearing their offspring, rather than prior to the onset of a breeding event [61]. However, our data showed that females may be intermediate between income and capital breeders, with a decreasing body condition index on both islands while rearing their chicks (Table 1). Both parents invest heavily in parental care and share incubation and chick-rearing responsibilities [29]. The difference between sexes in the alteration of body condition could be explained by the carry-over effect of egg formation in females. However, greater foraging effort by females while rearing the chick has also been observed [33], which may explain this, although the two factors are not mutually exclusive. Weight loss due to fasting during moulting in King penguins *Aptenodytes patagonicus* has been shown to increase δ^15^N by ~0.3 ‰ [25]. However, the extent of weight loss detected here for the breeding season (10%) was far less than that recorded previously for the moult (up to 40%; [62]). Therefore, we do not expect any important consequences of weight loss on the δ^15^N of females during chick-rearing.

The breeding cycle of African penguins is protracted and on Bird Island egg laying mainly takes place from February to July, sometimes followed by second clutches for both successful and failed breeders [29, 63]. When sampling adults in mid-January on Bird Island, no breeding activity had been recorded. However, considering their breeding cycle, laying in at least some individuals would likely have occurred shortly after sampling. The lower δ^13^C and elevated C:N ratios observed in female blood at that time of the year were therefore possibly related to higher proportions of circulating lipids in their blood due to egg formation [64]. Elevated lipid contents have been identified in the plasma of pre-breeding female Magellanic (*Spheniscus magellanicus* [65]) and Macaroni (*Eudyptes chrysolophus* [66]) penguins when compared to males and samples collected from females at other times of the year. No study has yet concurrently analysed stable isotopes and blood parameters of sexed penguins at the onset of laying. Measuring carbon and nitrogen stable isotope ratios of whole blood do not usually require a pre-treatment [38, 67], but as noted by [38] and observed in our study, care needs to be taken in particular physiological situations such as the egg formation period in females.

### Factors influencing the trophic ecology of African penguin

One of our most important results is that parents are selectively favouring small pelagics when catching food for their chicks, but targeting squid for self-provisioning, as indicated by blood and feather stable isotope data. Such selective foraging has also been observed in the Yellow-eyed penguin *Megadyptes antipodes* [68].

Our results from feathers indicate that adults from both islands preyed upon a mix of small pelagics and squid prior to moulting. Blood samples suggested that, thereafter, the adults maintained a similar diet prior to breeding. Birds with a lower body condition than average in January exhibited higher δ^15^N than birds with a higher body condition at the same period. This effect of physiology on δ^15^N values [56] may artificially and slightly increase the quantity of squid determined by the mixing model prior to breeding. During breeding, however, the adults obtained most of their energy from squid but fed the blues primarily on small pelagics together with lower amounts of squid (with subtle difference between islands). These changes in the diet may be dictated by the different energetic needs of the moult and breeding seasons, but may also follow the seasonal availability of prey species. Small pelagics, whose energy contents are ~1.5 times higher than that of squid [69], were favoured when energetic needs were important (before breeding and for chick growth). In addition, while adults are able to efficiently use squid [29], the chicks do not seem to possess that ability [70]. Growth and nutritional stress have been found to impact stable isotope ratios in chicks [57, 58] (but see [19]). To reduce the impact of growth on δ^13^C and δ^15^N values, we sampled chicks as closely as possible to fledging (i.e. blues; >61 days old). The weights recorded (~3000 g) for Bird and St Croix island blues suggest that they were not experiencing nutritional stress as these approached the weight of hand-reared chicks [71]. It is therefore unlikely that physiology impacted much on their δ^13^C and δ^15^N values. The geographical difference observed, with St Croix chicks being fed a larger proportion of higher trophic level prey such as squid than their counterparts on Bird Island, may be explained by the greater availability of squid in the south west area of the bay [72] where breeders from St Croix are known to forage [33].

The availability of the prey species in the environment was likely to also have played a role in the seasonality that we observed in adult diet. Penguins that have been tracked exclusively during chick-rearing on both islands remained in an area within 44 km of their breeding colony [33]. Therefore, it is likely that they will need to locate food within that area during breeding. The availability of small pelagics and squid in Algoa Bay is regulated by the annual cycle of these species, including the event known as the “sardine run”. This migration is a 1000 km northward migration of clupeids up the east coast to KwaZulu-Natal during the austral winter [73]. During this event the abundance of small pelagics (especially sardine) decreases significantly in the area just north of Algoa Bay [74]. Adult penguins may turn to squid during this period. The chokka squid is found all along the coast of southern Africa but the south east coast is particularly rich and known to host important spawning grounds in inshore waters [72]. Gregarious spawning, peaking in early summer, makes chokka squid particularly vulnerable to predators [75]. This may explain the large contribution of squid in the diet of African penguin during their pre-moult foraging trip in October despite the return of small pelagics.

Carbon and nitrogen stable isotope values of egg membranes were similar for the two islands suggesting that females from Bird and St Croix islands had a similar diet prior to laying. Unfortunately, the non-convergence of the MixSIAR model prevented the determination of diet from δ^13^C and δ^15^N values. The non-convergence could be explained by a number of factors including (i) the use of an erroneous trophic discrimination factor, and/or (ii) the absence of the main prey species in the database targeted by females prior to laying. Only one study has determined the diet-egg membrane trophic discrimination factor in penguins (Gentoo penguin *Pygoscelis papua*; [76]). A study of captive African penguins would allow the calculation of a species-specific discrimination factor and would verify the suitability of Gentoo penguin data. Should the discrimination factors be similar, African penguin females would be targeting high amounts of a currently undetermined species.

### Spatial and temporal variations in the diet of African penguin

Breeding colonies of African penguins are located both in the warm Agulhas current bioregion on the east coast of South Africa, and the cold Benguela current on the west [8]. These currents are characterised by different oceanographic conditions. This, in addition to differences in the bathymetry surrounding the breeding colonies, will likely influence the trophic ecology of these true central place foragers as it does in other penguin species [77, 78].

The trophic ecology of the African penguin has been studied all along its breeding range using stomach contents since 1950s (Table 4). In Namibia, African penguins turned to Pelagic goby *Sufflogobius bibarbatus* after the collapse of small pelagic stocks due to overfishing in the 1970s (Table 4; [79]). On the west coast of South Africa, small pelagics have consistently been recovered in the stomach contents since the end of the 1980s (Table 4). In the Eastern Cape, small pelagics and squid were identified as the main prey species in varying proportions depending on months and years during the early 1980s [14]. More recently, small pelagics have dominated the stomach contents at both St Croix and Bird islands (Table 4) which could be related to the eastward displacement of sardines and anchovy since the mid-1990s [80, 81].

**Table 4:** Studies that focused on the African penguin diet along its breeding range. Colonies are listed from West to East along the southern African coast. Small pelagics consists out of anchovy and sardine.

Country	Area	Colony	Year	Dominant prey	References
**Namibia**	**North**				
		Walvis Bay	1957–1958	Small pelagics	[12]
	**Central**				
		Mercury Isl.	1980	Pelagic goby	[82]
		Mercury Isl.	1980	Pelagic goby, Squid	[83]^a^
		Mercury Isl.	1996–2009	Pelagic goby^1^	[79]
		Ichaboe Isl.	1980	Pelagic goby	[82]
		Ichaboe Isl.	1980	Squid, Pelagic goby	[83]^a^
		Halifax Isl.	1977–1979	Pelagic goby	[82]
		Halifax Isl.	1980	Squid	[83]^a^
		Possession Isl.	1977–1979	Pelagic goby	[82]
		Possession Isl.	1980	Squid	[83]^a^
**South Africa**	**Western Cape**				
		St Helena Bay	1953–1954	Small pelagics	[84]
		St Helena Bay	1954–1955	Small pelagics	[85]
		West coast	1954–1056	Small pelagics, Mackerel^2^	[86]
		Saldana bay	1977–1978	Small pelagics^3^	[87]
		Marcus Isl.	1980–1986	Small pelagics	[88] (includes data from [13])
		Marcus Isl.	1990	Small pelagics	[89]
		Jutten Isl.	1987–1989	Small pelagics	[89]
		Dassen Isl.	1991–2009	Small pelagics^4^	[8]
		Robben Isl.	1989–1992	Small pelagics	[90]
		Robben Isl.	2003	Small pelagics	[91]
		Robben Isl.	1989–2009	Small pelagics	[8]
		Boulders	2003	Small pelagics	[91]
		Dyer Isl.	1991–1997, 2008, 2009	Small pelagics	[8]
	**Eastern Cape**				
		St Croix Isl.	1976–1977	Study of squid remains only	[92]
		St Croix Isl.	1979–1981	Small pelagics	[14]
		St Croix Isl.	1996, 1999, 2006, 2009	Small pelagics^5^	[8]
		St Croix Isl.	2009–2010	Small pelagics	[93]
		Bird Isl.	1992, 1993, 1999, 2001, 2005–2009	Small pelagics	[8]
		Bird Isl.	2009–2010	Small pelagics	[93]

^a^% by number converted to % by mass by [8]

^1^Except in 2003 when mullet was dominant

^2^Squid present but not numbered

^3^% by number

^4^Except in 1997 when other species were dominant

^5^Except in 1996 when squid was dominant

Squid remains have been found regularly in African penguin stomach contents. However, their importance was often dismissed due to the higher retention time of squid beaks in the stomachs compared to otoliths [94], and the slower digestion time of squid compared to fish [15]. Our study, using stable isotopes, demonstrated the importance of nutrients originating from squid for adults at various stage of their annual cycle and stresses the need for similar studies at other colonies to get a better understanding of their diet.

Two other projects have analysed stable isotopes of feathers and blood from African penguins [95, 96]. Differences in the protocols used, however, make comparisons difficult. For example, the lower δ^13^C detected in feathers collected in the Eastern Cape colonies in 2008–2009 [95] and in 2012–2013 [96] compared to our data are likely due to the effect of pigmentation rather than changes in the marine environment. Black feathers were analysed in the prior study but melanin has been shown to lower carbon stable isotope values [97]. By contrast, while δ^15^N is not affected by melanin content [97], the values observed in 2013 seem higher than the ones from previous years. This may indicate a slightly lower importance of squid in the earlier period during pre-moult foraging trips [95, 96]. Blood samples collected from adults at the beginning of winter in 2009 and 2013 on Bird Island (Algoa Bay) showed similar carbon and nitrogen stable isotope values highlighting the importance of small pelagics and squid during the breeding season for the adults [95]. Alternatively, if prey choice remained consistent in 2009, 2012 and 2013, a shift in the isotopic values of their preferred prey and/or temporal shift in isotopic baselines could have given rise to this difference [98]. Countering this, the large scale spatial variation in feather stable isotope values detected by [95] along the southern African coast mirrored that which was found for Cape gannets [99] and African black oystercatchers *Haematopus moquini* [100]. This indicates that these changes probably resulted from the different oceanographic conditions, and thus different foraging habitats, rather than a difference in the African penguin diet during their pre-moult foraging trip.

### Implications for conservation

This study provides a greater understanding of the trophic ecology of the African penguin at their most important breeding sites and reveals the utility of indirect methods in studying the diet of seabirds. It highlighted the importance of chokka squid at a particular time of the year in the diet of adults. Previous research have emphasized the role of small pelagic fish and how the purse sein fishing industry may negatively impact African penguins [93, 101]. We suggest that squid and squid fisheries should also be considered, in particular within areas of the African penguin breeding range. Further work should also focus on other breeding localities to access trophic information using indirect markers that is not available through traditional stomach content analysis. Stomach content data is the only source of information on diet that has been considered thus far in African penguin population modelling (e.g. [102–104]). Considering that this data can only be obtained from breeding animals, and the degree of seasonal variability in diet shown by this study, we suggest that this is inadequate. For species of conservation concern such as the African penguin, an effort should be made to fully understand their trophic ecology, how this vary with time, location, age class, and how it may change in the future.

## Supporting Information

S1 Table Measurement errors of carbon and nitrogen stable isotope values determined using three in-house standards that have been calibrated against materials from the International Atomic Energy Agency. Within runs data are presented as the range of SDs (n = number of runs). Overall values among all runs are presented as mean ± SD (n = number of standard duplicates). -: values not used in calibration.
(DOCX)

S2 Table Tissue- and species- specific discrimination factors between penguins and their food estimated from captivity studies. (DOCX)

S3 Table Comparisons in morphometric measurements, body weights, and body condition indices (BCI) of African penguins between sexes and between islands. (DOCX)

S4 Table Carbon and nitrogen stable isotope values and C:N ratios for the five potential prey species included into the Bayesian mixing model MixSIAR. n: number of samples.
(DOCX)

S5 Table Stable isotope mixing model (MixSIAR) results with predicted diet proportions (5^th^ to 95^th^ percentiles and median values in parentheses) of each five potential prey species compared to δ^13^C and δ^15^N mixture values of the different groups of African penguins. (DOCX)
