# Supplementary material for: Reappraisal of the Trophic Ecology of One of the World’s Most Threatened Spheniscids, the African Penguin
Source: PLoS One. 2016 Jul 19;11(7):e0159402. doi: 10.1371/journal.pone.0159402 (PMC4951110; doi:10.1371/journal.pone.0159402)
Supplement: S1 Table — Within runs data are presented as the range of SDs (n = number of runs). Overall values among all runs are presented as mean ± SD (n = number of standard duplicates). -: values not used in calibration. (DOCX) [file pone.0159402.s001.docx]

**S1 Table. Measurement errors of carbon and nitrogen stable isotope values determined using three in-house standards that have been calibrated against materials from the International Atomic Energy Agency.** Within runs data are presented as the range of SDs (n = number of runs). Overall values among all runs are presented as mean ± SD (n = number of standard duplicates). -: values not used in calibration.
