# Supplementary material for: Reappraisal of the Trophic Ecology of One of the World’s Most Threatened Spheniscids, the African Penguin
Source: PLoS One. 2016 Jul 19;11(7):e0159402. doi: 10.1371/journal.pone.0159402 (PMC4951110; doi:10.1371/journal.pone.0159402)
Supplement: S2 Table — (DOCX) [file pone.0159402.s002.docx]

**S2 Table. Tissue- and species- specific discrimination factors between penguins and their food estimated from captivity studies.**

**References**

Barquete V, Strauss V, Ryan PG. Stable isotope turnover in blood and claws: A case study in captive African Penguins. Journal of Experimental Marine Biology and Ecology 2013; 448: 121-127. doi: 10.1016/j.jembe.2013.06.021

Cherel Y, Hobson KA, Hassani S. Isotopic discrimination between food and blood and feathers of captive penguins: implications for dietary studies in the wild. Physiological and Biochemical Zoology 2005; 78(1): 106-115. doi: 10.1086/425202

Mizutani H, Fukuda M, Kabaya Y. δ^13^C and δ^15^N enrichment factors of feathers of 11 species of adult birds. Ecology 1992; 73(4): 1391-1395. doi: 10.2307/1940684

Polito MJ, Fisher S, Tobias CR, Emslie SD. Tissue-specific isotopic discrimination factors in gentoo penguin (*Pygoscelis papua*) egg components: Implications for dietary reconstruction using stable isotopes. Journal of Experimental Marine Biology and Ecology 2009; 372: 106-112. doi: 10.1016/j.jembe.2009.02.014

Polito MJ, Abel S, Tobias CR, Emslie SD. Dietary isotopic discrimination in gentoo penguin (*Pygoscelis papua*) feathers. Polar Biology 2011; 34: 1057-1063. doi: 10.1007/s00300-011-0966-5

Quillfeldt P, McGill RAR, Masello JF, Poisbleau M, van Noordwijk H, Demongin L, Furness RW. Differences in the stable isotope signatures of seabird egg membrane and albumen – implications for non-invasive studies. Rapid Communications in Mass Spectrometry 2009, 23: 3632-3636. doi: 10.1002/rcm.4286
