# Supplementary material for: Reappraisal of the Trophic Ecology of One of the World’s Most Threatened Spheniscids, the African Penguin
Source: PLoS One. 2016 Jul 19;11(7):e0159402. doi: 10.1371/journal.pone.0159402 (PMC4951110; doi:10.1371/journal.pone.0159402)
Supplement: S3 Table — (DOCX) [file pone.0159402.s003.docx]

**S3 Table. Comparisons in morphometric measurements, body weights, and body condition indices (BCI) of African penguins between sexes and between islands.**
