# Supplementary material for: Reappraisal of the Trophic Ecology of One of the World’s Most Threatened Spheniscids, the African Penguin
Source: PLoS One. 2016 Jul 19;11(7):e0159402. doi: 10.1371/journal.pone.0159402 (PMC4951110; doi:10.1371/journal.pone.0159402)
Supplement: S4 Table — n: number of samples. (DOCX) [file pone.0159402.s004.docx]

**S4 Table. Carbon and nitrogen stable isotope values and C:N ratios for the five potential prey species included into the Bayesian mixing model MixSIAR.** n: number of samples.
