# Supplementary material for: Reappraisal of the Trophic Ecology of One of the World’s Most Threatened Spheniscids, the African Penguin
Source: PLoS One. 2016 Jul 19;11(7):e0159402. doi: 10.1371/journal.pone.0159402 (PMC4951110; doi:10.1371/journal.pone.0159402)
Supplement: S5 Table — (DOCX) [file pone.0159402.s005.docx]

**S5 Table. Stable isotope mixing model (MixSIAR) results with predicted diet proportions (5^th^ to 95^th^ percentiles and median values in parentheses) of each five potential prey species compared to δ^13^C and δ^15^N mixture values of the different groups of African penguins.**

|  | **Island** | **Season** | **Age class** | **Sex** | **Anchovy** | **Sardine** | **Red-eye round herring** | **Mackerel** | **Squid** |
| --- | --- | --- | --- | --- | --- | --- | --- | --- | --- |
| 1. **Blood** | |  |  |  |  |  |  |  |  |
|  | **Bird** | **Non-breeding** | **Adults** | **Males** | 0.01–0.35 | **0.19-0.60** | 0.01-0.36 | 0.00-0.10 | 0.22-0.36 |
|  |  |  |  |  | (0.09) | **(0.45)** | (0.08) | (0.02) | (0.29) |
|  |  |  |  | **Females** | 0.03–0.37 | **0.17-0.48** | 0.02-0.37 | 0.00-0.17 | 0.21-0.34 |
|  |  |  |  |  | (0.16) | **(0.33)** | (0.15) | (0.05) | (0.28) |
|  |  | **Breeding** | **Adults** | **Males** | 0.01-0.31 | 0.05-0.36 | 0.01-0.32 | 0.00-0.12 | **0.43-0.55** |
|  |  |  |  |  | (0.12) | (0.20) | (0.12) | (0.03) | **(0.49)** |
|  |  |  |  | **Females** | 0.03-0.29 | 0.08-0.32 | 0.02-0.29 | 0.00-0.14 | **0.42-0.55** |
|  |  |  |  |  | (0.13) | (0.19) | (0.13) | (0.04) | **(0.48)** |
|  |  |  | **Blues** |  | 0.02-0.41 | **0.13-0.52** | 0.01-0.36 | 0.00-0.11 | 0.25-0.37 |
|  |  |  |  |  | (0.15) | **(0.35)** | (0.12) | (0.02) | (0.31) |
|  | **St Croix** | **Breeding** | **Adults** | **Males** | 0.01-0.31 | 0.07-0.39 | 0.01-0.31 | 0.00-0.09 | **0.42-0.54** |
|  |  |  |  |  | (0.11) | (0.25) | (0.11) | (0.02) | **(0.48)** |
|  |  |  |  | **Females** | 0.02-0.28 | 0.09-0.34 | 0.02-0.28 | 0.00-0.10 | **0.42-0.55** |
|  |  |  |  |  | (0.12) | (0.21) | (0.12) | (0.03) | **(0.49)** |
|  |  |  | **Blues** |  | 0.01-0.34 | 0.11-0.46 | 0.01-0.32 | 0.00-0.09 | **0.36-0.48** |
|  |  |  |  |  | (0.10) | (0.31) | (0.09) | (0.02) | **(0.42)** |
| 1. **Feathers** | |  |  |  |  |  |  |  |  |
|  | **Bird** | **Non-breeding** | **Adults** | **Males** | 0.00-0.29 | **0.17-0.51** | 0.00-0.29 | 0.00-0.09 | **0.35-0.51** |
|  |  |  |  |  | (0.07) | **(0.36)** | (0.07) | (0.01) | **(0.43)** |
|  |  |  |  | **Females** | 0.01-0.27 | **0.19-0.49** | 0.01-0.28 | 0.00-0.09 | **0.33-0.48** |
|  |  |  |  |  | (0.08) | **(0.36)** | (0.08) | (0.02) | **(0.40)** |
|  |  | **Breeding** | **Adults** | **Males** | 0.00-0.23 | **0.27-0.55** | 0.01-0.25 | 0.00-0.07 | **0.32-0.46** |
|  |  |  |  |  | (0.05) | **(0.43)** | (0.07) | (0.01) | **(0.39)** |
|  |  |  |  | **Females** | 0.01-0.24 | **0.24-0.50** | 0.01-0.26 | 0.00-0.10 | **0.31-0.47** |
|  |  |  |  |  | (0.08) | **(0.37)** | (0.10) | (0.02) | **(0.39)** |
|  |  |  | **Blues** |  | 0.00-0.22 | **0.50-0.78** | 0.00-0.24 | 0.00-0.08 | 0.10-0.26 |
|  |  |  |  |  | (0.05) | **(0.66)** | (0.05) | (0.01) | (0.18) |
|  | **St Croix** | **Breeding** | **Adults** | **Males** | 0.01-0.28 | **0.21-0.51** | 0.01-0.29 | 0.00-0.09 | **0.32-0.46** |
|  |  |  |  |  | (0.09) | **(0.36)** | (0.09) | (0.02) | **(0.39)** |
|  |  |  |  | **Females** | 0.01-0.26 | **0.21-0.49** | 0.01-0.27 | 0.00-0.11 | **0.31-0.48** |
|  |  |  |  |  | (0.09) | **(0.35)** | (0.10) | (0.03) | **(0.40)** |
|  |  |  | **Blues** |  | 0.00-0.19 | **0.49-0.77** | 0.00-0.20 | 0.00-0.07 | 0.12-0.30 |
|  |  |  |  |  | (0.04) | **(0.64)** | (0.05) | (0.01) | (0.21) |
| 1. **Egg membrane** | | |  |  |  |  |  |  |  |
|  | **Bird** |  |  |  | x | x | x | x | x |
|  | **St Croix** |  |  |  | x | x | x | x | x |

Values in bold are the highest prey species contribution. ‘x‘: the model did not converge.
